# Management of Metastatic Endometrial Cancer: Physicians’ Choices Beyond the First Line. A MITO Survey

**DOI:** 10.3389/fonc.2022.880008

**Published:** 2022-05-27

**Authors:** Gaia Giannone, Daniele Castaldo, Valentina Tuninetti, Giulia Scotto, Margherita Turinetto, Anna Amela Valsecchi, Michele Bartoletti, Serafina Mammoliti, Grazia Artioli, Giorgia Mangili, Vanda Salutari, Domenica Lorusso, Gennaro Cormio, Claudio Zamagni, Antonella Savarese, Massimo Di Maio, Graziana Ronzino, Carmela Pisano, Sandro Pignata, Giorgio Valabrega

**Affiliations:** ^1^ Candiolo Cancer Institute, Fondazione del Piemonte per l'Oncologia (FPO) - IRCCS, Candiolo, Italy; ^2^ Department of Oncology, University of Turin, Turin, Italy; ^3^ Department of Surgery and Cancer, Imperial College London, London, United Kingdom; ^4^ Segreteria Multicenter Italian Trials in Ovarian Cancer and Gynecologic Malignancies (MITO) Group, Naples, Italy; ^5^ Unit of Medical Oncology and Cancer Prevention, Department of Medical Oncology, Centro di Riferimento Oncologico di Aviano (CRO), IRCCS, Aviano, Italy; ^6^ Ospedale Policlinico San Martino - Department of Medical Oncology 1- L.go Rosanna Benzi, IRCCS, Genoa, Italy; ^7^ Oncologia Medica, Unità locale socio sanitaria n2 (ULSS2) Marca Trevigiana, Treviso, Italy; ^8^ Obstet-Gynecol Department, San Raffaele Scientific Institute, IRCCS, Milan, Italy; ^9^ Department of Women and Child Health, Division of Gynecologic Oncology, Fondazione Policlinico Universitario A. Gemelli, IRCCS, Rome, Italy; ^10^ Department of Life Science and Public Health, Catholic University of Sacred Heart Largo Agostino Gemelli, and Fondazione Policlinico Universitario A. Gemelli, IRCCS, Rome, Italy; ^11^ Department of Interdisciplinary Medicine (DIM), University of Bari, Bari, Italy; ^12^ Azienda Ospedaliero-universitaria di Bologna, IRCCS, Bologna, Italy; ^13^ Department of Oncology, Regina Elena National Cancer Institute, Rome, Italy; ^14^ Department of Oncology, Mauriziano Hospital, University of Turin, Turin, Italy; ^15^ Department of Oncology, Ospedale “Vito Fazzi”, Lecce, Italy; ^16^ Department of Urology and Gynecology, Istituto Nazionale Tumori IRCCS Fondazione G. Pascale Napoli, Naples, Italy

**Keywords:** endometrial cancer, molecular classification, second line therapy, immune checkpoint inhibitors, MSI, survey

## Abstract

**Background:**

Endometrial cancer (EC) therapeutic and diagnostic approaches have been changed by the development of a new prognostic molecular classification, the introduction of dostarlimab in microsatellite instability (MSI) high pre-treated advanced EC patients with further expected innovation deriving from lenvatinib plus pembrolizumab regardless MSI status. How this is and will be translated and embedded in the clinical setting in Italy is not known; this is why we developed Multicentre Italian Trials in Ovarian cancer and gynaecologic malignancies (MITO) survey on the current practice and expected future changes in EC.

**Methods:**

We designed a self-administered, multiple-choice online questionnaire available only for MITO members for one month, starting in April 2021.

**Results:**

75.6% of the respondents were oncologists with a specific focus on gynaecologic malignancies and 73.3% of the respondents declared the availability of clinical trials in second line treatment for advanced EC. The therapeutic algorithm in second line was heterogeneous, being the most frequent choice administering anthracyclines followed by endocrine therapy or enrolling in clinical trials. While more than half of the clinicians declared that they performed the molecular classification, only six/45 respondents (13.3%) ran all the tests needed for it. On the other hand, 80% of them declared regular assessment of MSI status with IHC as recommended. The therapeutic approach in MSI high advanced EC patients has changed since dostarlimab approval. Indeed the most frequent choice in second line has been chemotherapy (53.3%) before its availability, while dostarlimab has been preferred in more than three-fourths of the cases (75.6%) after its approval. As for MSS patients, 77.8% of clinicians would choose lenvatinib plus pembrolizumab for them in second line once approved.

**Conclusions:**

Despite the selected sample of respondents from Italian MITO centres showing good knowledge of diagnostic and therapeutic innovations in EC, these are not fully implemented in everyday clinics, except for MSI status assessment.

## Introduction

In 2021, more than 400,000 new diagnoses of endometrial cancer (EC) have been estimated worldwide (1–3). Most of the new cases are early-stage malignancies because one of the most frequent symptoms, vaginal bleeding, is extremely precocious leading to early diagnosis with overall survival at 5 years of 81.1% (1, 2).

Nonetheless, patients with advanced and recurrent disease have a dismal prognosis with an expected 5-year survival of less than 20% and scarce treatment options (4). Indeed, patients with metastatic disease are candidates for a platinum-based chemotherapy with an expected median progression-free survival (PFS) of 13 months, while in second and further lines few studies are available and monotherapy with anthracyclines as well as platinum rechallenge, weekly paclitaxel, or endocrine therapy are usually the preferred choices, with low chances of response (4–6).

During the last years, both the diagnostic and therapeutic scenarios have changed dramatically in this field. From a diagnostic point of view, we overcame the traditional two-types classification based on Bokhman’s clinical, metabolic, and endocrine features to a molecular and pathological driven definition of risk groups (7–10). Four subgroups have been identified by The Cancer Genome Atlas (TCGA) according to molecular features. An ultramutated group with frequent DNA Polymerase Epsilon (POLE) exonuclease mutations and a good prognosis, a hypermutated group with Microsatellite instable (MSI) cancers, harbouring a Mismatch repair deficiency (MMRd), a copy number low group, including most of the microsatellite stable (MSS) endometrioid cancers, and a serous-like group with frequent

Tumor Protein P53 (TP53) mutations (10). In addition to the prognostic role of this classification, it might help drive therapeutic choices. Specifically, serous-like tumours have the worst prognosis and are characterized by a low immune infiltrate while POLE and MSI cancers are characterized by a high predicted neo-antigens load, overexpression of PD-1 and PD-L1, and massive CD3+ and CD8+ Tumour-associated lymphocytes infiltration, thus suggesting that these two subgroups might be the best candidates for immunotherapy (9–11). Several studies independently demonstrated that the diagnostic algorithm can be implemented using a few immunohistochemical markers [p53, MutS Homolog 6 (MSH6), and PMS1 Homolog 2 (PMS2), at least, though the gold standard is the assessment of the four MMR proteins: MutL Homolog 1 (MLH1), MutS Homolog 2 (MSH2), MSH6, and PMS2), and only one molecular test (mutation analysis of the hotspots in the exonuclease domain of POLE) to identify prognostic groups, which mostly overlap the TCGA molecular-based classification (12–17). These studies did not only show the feasibility of this approach but also confirmed the prognostic role of this classification, above all in early-stage EC (12–17). Of note, to classify an EC sample according to this molecular classification all the diagnostic tests described above need to be performed (4). Up to now, the molecular classification plays an important role in the choice of adjuvant treatment, and it is recommended, when feasible, by the new ESMO-ESGO-ESTRO Guidelines in all early-stage EC (4). Moreover, the universal screening for MSI/MMR status is of uppermost importance, since it is the first step to find patients and thereafter relatives (healthy carriers) with Lynch Syndrome (18, 19). In these healthy carriers, genetic counselling and an intensified follow-up is recommended to detect malignancies at an early stage (18). On the other hand, the therapeutic role of this classification in late disease has been explored in the last few years, with the beginning of the immunotherapy era also in EC. Indeed, for patients with MMRd tumours, the current treatment algorithm in advanced disease has been revolutionized by the introduction of checkpoint inhibitors (20, 21). First pembrolizumab and then dostarlimab, with a large phase Ib trial, demonstrated activity in patients with MMRd tumours (20–22). Specifically, 104 patients received dostarlimab as a single agent in second or further lines with an objective response rate of 42.3%, including 12.7% confirmed complete response and a median duration of response which was not reached at a median follow-up of 11.2 months (21). This lead to the approval of dostarlimab by the U.S. Food and Drug Administration (FDA) and received conditional marketing authorisation by European Medicines Agency (EMA), thus being available in Italy within an expanded access program in January 2021 (23, 24). A further reshaping of the treatment algorithm is expected also in patients without MMRd tumours after the release of Study 309/KEYNOTE-775 results, a phase III trial conducted in patients pre-treated with a platinum doublet, showing improvement in terms of PFS and overall survival (OS) with the combination of pembrolizumab and lenvatinib, compared with a standard treatment irrespective of MSI status, with a manageable safety profile (25, 26).

How much of this knowledge has been transferred and is available in Italian everyday diagnostic and therapeutic algorithms is not known as well as we cannot predict if and how much the new combination of lenvatinib and pembrolizumab would be the chosen regimen for EC patients. Therefore, we led a survey among Multicenter Italian Trials in Ovarian cancer and gynaecologic malignancies (MITO) centres to evaluate the current management in EC, how the new discoveries have impacted the daily clinical practice, and the expected changes across Italy in 2021. The main objective of the investigation was to evaluate current practice in EC among different centres.

## Methods

We developed a survey which was a self-administered online questionnaire. The survey was developed by GG and GV, reviewed and discussed by the MITO scientific committee; submitted to and approved by the MITO internal review board. Thereafter, it was available on the MITO website only for MITO members from April 12, 2021 to May 7, 2021. Specifically, the survey was composed of 25 multiple choice questions (see the list of questions in the **Supplementary Table S1**). The first nine questions focused on the characteristics of the respondents and on the number of patients treated in each centre; nine questions dealt with the therapeutic algorithm in second line (and how it changed or was expected to change due to the introduction of immune checkpoint inhibitors), and six with the diagnostic algorithm, while one question asked about COVID19 impact in this setting. We analysed one answer form per each centre. All replies were anonymized. Descriptive analyses are detailed in the results session.

## Results

An invitation to complete the survey was sent to 691 MITO members, for a total of 175 centres. Among them, 284 clinicians (41.1%) opened the invitation, 52 (7.5%) clicked on the link, and 49 (7.1%) completed the survey. In three cases, more than one respondent per centre was recorded and we analysed only one questionnaire per centre. A total of 45 responses (25.7% of the MITO centres) were therefore analysed. Most of the respondents were aged 40 or more (34/45, 75.6%) and worked in a public hospital (17/45, 37.8%) or university hospital (15/45, 33.3%). More than 75% of the respondents (34/45) treated mainly but not exclusively patients with gynaecological cancers, being most of the questionnaires completed by medical oncologists (34/45, 75.6%) (see **Table 1**). The physicians completing the survey were well distributed across the country with 20 of them (44.4%) working in hospitals located in the North of Italy while 15 (33.3%) and 10 (22.2%) were from the Centre and the South of Italy respectively (see **Table 1**). Most of the responders had a medium volume of EC patients. Indeed 25 (55.6%) clinicians had 5 to 10 new diagnoses of EC per month with seven (15.6%) and six (13.3%) of them treating 11 to 25 and more than 25 new cases of EC, respectively, per month. More than half of the respondents (24/45, 53.3%) treated 5 to 10 advanced or metastatic EC patients per month with 16 (35.6%) and 5 (11.1%) of them seeing in everyday clinic less than five patients and more than 10 patients, respectively. In second and further lines, the volume is similar, with 22 (48.9%) physicians seeing five to 10 EC patients in this setting per month while 15 (33.3%) and eight (17.8%) respondents treated less than five patients per month and more than 10 per month, respectively.

**Table 1:** Respondents’ characteristics.

Respondents characteristics		
Feature	Number	Percentage
**Age**		
**<40 years old**	11	24,4
**>40 years old**	34	75,6
**Years in practice (focus on gynaecological cancer)**	14,8 years (average)	
**Health organizations where the respondents work**		
**Public hospital**	17	37,8
**University Hospital**	15	33,3
**Istituto Di Ricovero e Cura a Carattere Scientifico (Italian institutes for research and care)**	10	22,2
**Private Hospital**	2	4,4
**Other**	1	2,2
**Location of the Hospital**		
**North of Italy**	20	44,4
**Centre of Italy**	15	33,3
**South of Italy, Sicily or Sardinia**	10	22,2
**Medical training**		
**Medical Oncology**	34	75,6
**Gynaecology**	10	22,2
**Other**	1	2,2
**Clinical focus**		
**Only gynaecological cancers**	9	20,0
**Mainly gynaecological cancers**	34	75,6
**Other**	2	4,4
**Cumulative number of new EC diagnoses per month**		
**Less than 5**	7	15,6
**5-10**	25	55,6
**11-25**	7	15,6
**More than 25**	6	13,3
**Cumulative number of recurrent, locally advanced (unresectable) or metastatic EC patients treated per month**		
**Less than 5**	16	35,6
**5-10**	24	53,3
**More than 10**	5	11,1
**Cumulative number of pretreated metastatic EC patients treated per month**		
**Less than 5**	15	33,3
**5-10**	22	48,9
**More than 10**	8	17,8

a EC, endometrial cancer.

**Figure 1:**
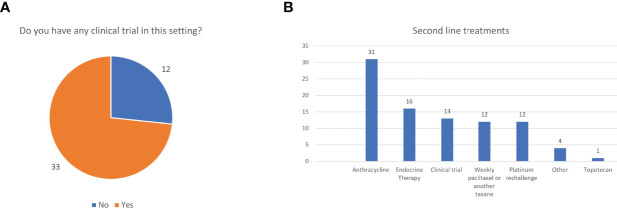
Clinical trials **(A)** and treatment choices **(B)** in second line.

**Figure 2:**
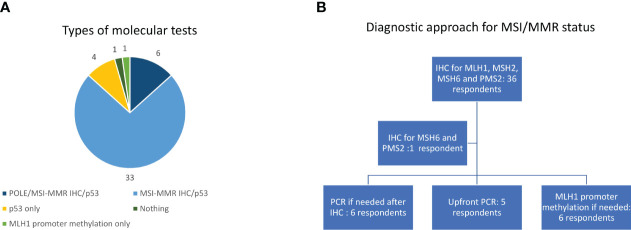
Types of molecular tests **(A)** and diagnostic approach for MSI/MMR status **(B)**. IHC, immunohistochemistry; MMR, mismatch repair; MSI, Microsatellite instability; PCR, Polymerase chain reaction.

More than 75% of patients received second line treatment in the experience of 23 (51.1%) of them while 20 (44.4%) respondents offered second line treatment to 50%-75% of their EC patients. The most frequent reasons for not proposing an active treatment were frail general conditions in 22 (48.8%) and a combination of comorbidities and bad performance status in 16 (35.6%) cases while two (4.4%) clinicians said they did not candidate patients to second line because of the absence of effective treatments. Thirty-three respondents (73.3%) confirmed the availability, for patients treated at their institution, of clinical trials in this setting, while 12 (26.7%) did not (**Figure 1A**). We asked which were the preferred treatments (requiring a maximum of two answers). The drugs administered in second line were extremely heterogeneous in our cohort being the most frequent choices anthracyclines (31 cases, 68.9%), endocrine therapy (16 cases, 35.6%), enrolment in a clinical trial (13 cases, 28.9%), weekly paclitaxel (or another taxane), or a rechallenge with platinum (12, respondents, 26.7%, each) (**Figure 1B**). Nearly all the responders confirmed that they evaluated hormonal receptor (oestrogen and or progesterone receptors) (42/45, 93.3%) using immune histochemistry (IHC) while 25 (55.6%) of them said that they performed the molecular classification in their centre. Nevertheless, 6/45respondents (13.3%) ran all the tests needed for it (POLE hotspots sequencing, IHC for MMR proteins or MSI status defined using polymerase chain reactions -PCR- and p53 IHC). Thirty-three of 45 respondents (73.3%) evaluated p53 and MMR proteins using IHC, being p53 IHC the only performed test for four interviewees (13.3%) (**Figure 2A**).

The most frequent approach to evaluate MSI/MMR status was IHC (36 cases, 80%) for all the four proteins (MLH1, MSH2, MSH6, PMS2) with one respondent (2.2%) evaluating only MSH6 and PMS2 (**Figure 2B**). Six clinicians (13.3%) used PCR as a second step approach for indeterminate cases at IHC while it was performed upfront in five cases (11.1%) (**Figure 2B**). Only six respondents (13.3%) evaluated MLH1 methylation status (**Figure 2B**). We asked in which moment of the patient journey MSI/MMR status was assessed, and 33 clinicians (73.3%) responded that it was screened universally in every patient with a new diagnosis of EC while it was evaluated in second or further lines to define the best treatment choice in eight cases (17.8%). Once a deficiency in MMR machinery was detected on the tumour specimen, genetic counselling was planned before the blood sampling for the germline testing in 22 centres (48.9%), after the germline confirmation of a Lynch Syndrome in eight centres (17.8%) and in patients with both confirmed germline MMRd or a high suspect of Lynch Syndrome according to their family history in six centres (13.3%). Only eight respondents (17.8%) said they referred for genetic counselling all EC patients with a family history suspicious for Lynch Syndrome even before testing MSI/MMR status on the tumour sample.

**Figure 3:**
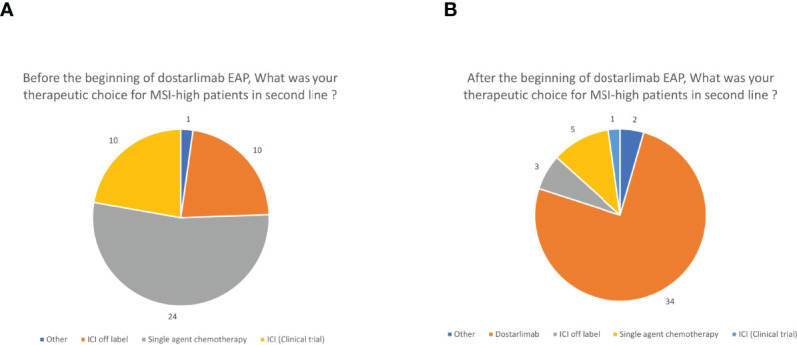
Therapeutic choices before **(A)** and after **(B)** the beginning of dostarlimab expanded access program (EAP). ICI, immune checkpoint inhibitor other than dostarlimab.

The therapeutic approach in MMRd patients has been changed according to the respondents in the last year with the availability of the expanded access program of dostarlimab (**Figure 3**). Indeed, before its availability, most of them (24, 53.3%) treated patients with a single agent chemotherapy in second line while 20 out of 45 (44.4%) proposed a checkpoint inhibitor off-label, paid by the hospital, or a clinical trial (10 respondents each, 22.2%) (**Figure 3A**). Since dostarlimab approval, 34 respondents (75.6%) think that it is the best option for MMRd EC; only five respondents (11.1%) are continuing to administer a monotherapy with another cytotoxic agent in this setting, and the remaining respondents are preferring a checkpoint inhibitor off-label (3,6.7%), a clinical trial (1, 2.2%) or other treatments (2, 4.4%) (**Figure 3B**). During the 5 months of dosarlimab availability, 13 clinicians (28.9%) said they have never prescribed dostarlimab and 21 (46.7%) had no patients on treatment with dostarlimab while 11 (24.4%) clinicians had one to five patients receiving dostarlimab at time of the survey. This new drug has changed the MSI/MMR status screening only in 20 (44.4%) cases, with the introduction of this test in the advanced setting. No changes were declared from the remaining respondents because there was a universal screening system before dostarlimab availability (19 cases, 42.2%) or because it continued to be proposed in selected cases (5 cases, 11.1%).

As for MMR proficient (MMRp) patients, 35/45 clinicians (77.8%) affirmed that the combination of lenvatinib plus pembrolizumab, according to KEYNOTE-775 results, was going to become the preferred choice for the second line setting, when available.

Lastly, we asked how COVID-19 pandemic impacted EC management with 33 interviewees (73.3%) saying it did not impact at all on the treatment of EC patients; 12 (26.7%) clinicians responded that they modified the follow-up (longer interval and/or phone calls instead of in-clinic visits) while no difference was recorded in treatment indications or administration.

## Discussion

This survey is a snapshot of the diagnostic and therapeutic choices for advanced pre-treated EC in Italian MITO centres. It highlights how the new molecular classification has not been extensively implemented in the clinical setting. Moreover, it confirms that the therapeutic approach beyond first line is extremely heterogeneous. Indeed, since its availability, dostarlimab has been the preferred choice for MMRd patients but, probably for the short timespan between its approval and our survey and the low number of patients with pre-treated MMRd EC, a small number of women were receiving or had received this treatment at the time of the survey, with more than one-fourth of the respondents having never prescribed it.

An important point that should be underlined regards the features of the interviewed population. We administered this questionnaire *via* the official web site and newsletter of MITO group, which involves centres with a focus on gynaecological cancer and who are keen to enroll gynaecological patients in clinical trials. However, only around 25% of the MITO centres responded to this survey and most of the responses were from medical oncologists.

This implies a possible selection bias and makes it difficult to generalize our results to all Italian hospitals but, on the other hand, the respondents were well distributed across the country, most of them with a long experience and a medium to high volume of EC patients, being a low number of them focused only on gynaecological malignancies. We believe that this is the most frequent setting in which a woman with a relapsed EC is treated or to which she is referred.

Most of the EC patients were candidates to second line of treatment and the reasons not to propose a further treatment are usually comorbidities instead of an expected lack of benefit from drugs administered in pre-treated women (27). The response rate in this setting is lower than 20% but, on the other hand, the availability of clinical trials in nearly three-fourth of the centres suggests once again that there are more therapeutic options for these hospitals and that the positive attitude toward administering experimental treatments is extremely solid (5). The heterogeneity of drugs prescribed in second or further lines is concordant with the literature, in the absence of head-to-head comparisons between single agents or between chemotherapy and endocrine therapy, with the last one being the preferred option in grade 1 slow progressing EC (4, 5, 28–30). Interestingly, our results are similar to a German survey in which chemotherapy was preferred to progestins, although a wide variability in the choices was recorded (31).

In our survey, most of the centres performed oestrogen and progesterone receptor assessment which has a prognostic role but does not drive therapeutic choices (32). On the other hand, slightly more than half of the interviewees stated that they have implemented the EC molecular classification in clinical practice. Surprisingly though, only in six hospitals, all the required diagnostic tests are run together leading to two conclusions (4). The first one is that we are far from the optimal setting in which treatment decisions can be driven by an accurate assessment of molecular characteristics of each EC, being difficult and expensive to implement it also in dedicated settings such as the MITO centres. The second one is that we probably need to increase the knowledge on how the molecular classification is performed, perhaps supporting educational meetings with pathologists and lab researchers, being a field in which the well-known IHC is side-by-side to novel sequencing techniques (PCR and hotspot sequencing) (4). On the other hand, universal screening for Lynch Syndrome is performed by more than three-quarters of the respondents as suggested by international and national guidelines but only six of them have appropriate facilities performing MLH1 promoter methylation assessment, thus reducing the number of unnecessary genetic referrals (4, 19, 33). Moreover, the timing for the genetic referral is quite variable though around half of the interviewees refer patients right after the assessment of MSI/MMR status on tumour specimens.

How both diagnostic and therapeutic implementations reflect into the treatment choices is quite impressive. Before the availability of dostarlimab, most of the clinicians administered a cytotoxic agent also to MMRd patients in second line, although around 40% of them had the possibility to propose an immune checkpoint inhibitor (off-label or in the setting or a clinical trial). After the beginning of the expanded access program, more than one-fourth of them are choosing to prescribe dostarlimab. Notwithstanding, a low number of patients have been treated with this drug so far, which is probably due to the rarity of the setting and the short timespan between the approval and the end of the survey.

It is moreover expected a change in the therapeutic algorithm also in MMRp patents, with nearly 80% of the respondents believing that the preferred treatment in this setting will be lenvatinib plus pembrolizumab which has been approved by EMA in December 2021 regardless MMR status.

Lastly it seems that COVID-19 had little effect on therapeutic management of EC patients. Previous surveys suggested that the pandemic impacted the treatment choices above all focusing on ovarian cancer patients, thereafter it would be interesting to record and evaluate EC patient outcomes during these years in which, on one hand, new therapeutic options are available after decades but, on the other, the challenge of a global threaten is faced, redirecting resources for research and treatment to this emergency (34, 35).

Our study has several limitations; the most important ones are the possible selection biases deriving from the low number of MITO members who filled in the questionnaires, with feedbacks from one-fourth of the MITO centres. Moreover, the interviewed centres have a focus on gynaecological malignancies and there was prevalent participation of oncologists, while the treatment of these women is carried out by both gynaecologists and oncologists in Italy. As for the questionnaire, to avoid heterogeneity, we chose closed-ended questions in most of the cases, which do not allow to represent the various nuances of the therapeutic and diagnostic pathways.

In addition, these results are too premature to evaluate and weight the changes in treatment for MMRd EC and the survey was available only for one month. We are expecting, in view of the answers collected, that the therapeutic scenario will be improved for all patients with advanced EC and that a better classification of early ones will allow us to personalize the adjuvant treatment and further reduce the risk of recurrence. This is why a follow-up survey will be administered to all MITO members with the aim of evaluating if there has been an improvement, with better knowledge and wider availability of these tools in the clinical setting over the last year. How these changes will impact the quality of life and survivorship of women who have usually important comorbidities is not known. It is, indeed, of uppermost importance to plan real-life studies which will evaluate if there is an implementation of the molecular assays in these centres, how dostarlimab treatment is managed, which are the long-term outcomes and toxicities, and if there is any impairment in quality of life.

## Data Availability Statement

The raw data supporting the conclusions of this article will be made available upon request by the authors, without undue reservation.

## Author Contributions

Authors’ contributions: GG and GV: Conceptualization; all authors: resources, GG and DC: Data curation; GG and GV: Formal analysis, Software and Methodology; GV: Funding acquisition; GG and GV: Investigation and Project administration; GV: Supervision, Validation and Visualization; GG and GV: drafting of the manuscript; GG, GV, MDM, and DL: review & editing; all authors: final approval of the version to be published.

## Funding

This article was partially funded by VALG_RILO_20_01 to GV.

## Conflict of Interest

GG received a grant from ESMO and payment for educational events from Mylan, she coordinates MITO Gruppo Formazione. DL received grants or contracts from GSK, MSD, Clovis Oncology, consulting fees from Pharmamar, Merck Serono, payment or honoraria for lectures, presentations, speakers bureaus, manuscript writing or educational events from GSK, Clovis Oncology, Astra Zeneca, MSD; payment for expert testimony from Clovis Oncology; support for attending meetings and/or travel from GSK, Roche, Pharmamar; participation on a Data Safety Monitoring Board or Advisory Board for Novartis, Seagen, MSD, Astra Zeneca, Immunogen, Genmab, Amgen, Clovis Oncology, GSK, Merck Serono and she is Chair of Gynecological Cancer Accademy, Bord of Director of Gynecological cancer Integroup. MDM received Grants or contracts to his institution from Tesaro and GSK, consulting fees from Novartis, Roche, AstraZeneca, Merck Serono, Pfizer, Merck Sharp & Dohme, Janssen, Eisai, Takeda, Boehringer Ingelheim, Servier; Payment or honoraria for lectures, presentations, speakers bureaus, manuscript writing or educational events from Novartis, Roche, AstraZeneca, Pfizer, Merck Sharp & Dohme, Janssen, Astellas, Boehringer Ingelheim; Participation on a Data Safety Monitoring Board or Advisory Board for Merck Sharp & Dohme, Janssen, Astellas and Amgen.

The remaining authors declare that the research was conducted in the absence of any commercial or financial relationships that could be construed as a potential conflict of interest.

## Publisher’s Note

All claims expressed in this article are solely those of the authors and do not necessarily represent those of their affiliated organizations, or those of the publisher, the editors and the reviewers. Any product that may be evaluated in this article, or claim that may be made by its manufacturer, is not guaranteed or endorsed by the publisher.

## Abbreviations

CD3, Cluster of differentiation 3; CD8, Cluster of differentiation 8; EC, Endometrial Cancer; IHC, Immune Histochemistry; MITO, Multicenter Italian Trials in Ovarian cancer and gynecologic malignancies; MLH1, MutL Homolog 1; MMRd, Mismatch Repair Deficiency; MSH2, MutS Homolog 2; MSH6, MutS Homolog 6; MSI, Microsatellite Instability/Instable; MSS, Microsatellite stable; PCR, polymerase chain reaction; PD-1, Programmed cell death protein 1; PDL-1, Programmed death-ligand 1; PFS, Progression Free Survival; PMS2, PMS1 Homolog 2; POLE, DNA Polymerase Epsilon; PTS, patients; TCGA, The Cancer Genome Atlas; TP53, Tumor Protein P53.
