# Supplementary material for: Management of Metastatic Endometrial Cancer: Physicians’ Choices Beyond the First Line. A MITO Survey
Source: Front Oncol. 2022 May 27;12:880008. doi: 10.3389/fonc.2022.880008 (PMC9185800; doi:10.3389/fonc.2022.880008)
Supplement: Supplementary file 1 [file Table_1.docx]

| **Questions:** |
| --- |
| How old are you? |
| For how many years you have been working in the setting of gynecology oncology? |
| In what type of Hospital do you work? |
| In which area of Italy do you work? |
| Which is your speciality? |
| Do you manage above all gynaecologic malignancies/ only gynaecologic malignancies/other cancer types and marginally gynaecologic malignancies? |
| How many new Endometrial cancers (EC) are diagnosed in your centre in one month? |
| How many patients with recurrent/locally advanced not amenable for surgical treatment/metastatic EC are managed in your centre in one month? |
| How many patients with pretreated metastatic EC are managed in your centre in one month? |
| Which is the average ECOG Performance status of the patients treated in second (II) line? |
| What percentage of pretreated EC patients are candidates to II line treatment? |
| Which are the most frequent reasons for not proposing II line treatments in pretreated advanced EC patients in your experience? |
| Do you have any clinical trial in this setting? |
| Which are the most frequent II line treatments in these patients in your experience (maximum 2 choices)? |
| Do you perform Estrogen/Progesterone Receptor immunohistochemistry (IHC) for EC patients’ samples? |
| Do you use the molecular classification for EC patients in everyday practice? |
| Which assays are performed at diagnosis in EC patients in your centre? (Among IHC for MSH6 and PMS2; IHC for MLH1, MSH2, MSH6 and PMS2; Polymerase chain reaction (PCR) for MSI; IHC for p53, POLE hotspot sequencing; MLH1 promoter methylation or others) |
| In which setting do you perform this test? |
| Which was your preferred treatment in II line for advanced Mismatch repair deficient (MMRd) patients before dostarlimab approval? |
| Which is/will be your preferred treatment in II line for advanced MMRd patients since dostarlimab approval? |
| How many patients are receiving dostarlimab in your centre now? |
| In view of KEYNOTE-775 results do you think that Pembrolizumab/Lenvatinib is going to be the preferred choice in II line for Mismatch repair proficient (MMRp) advanced EC patients? Why? |
| Did COVID 19 pandemic affect the therapeutic approach in EC patients in your patients? |
| Did you change the diagnostic algorithm in your centre after dostarlimab approval? |
| Which are the subsequent steps after a diagnosis of MMRd EC? How do you organize genetic referrals in your centre? |

Table S1: MITO questionnaire (translated into English).
